# 35.3 CHILDHOOD EXPOSURE TO GREEN SPACE – A NOVEL RISK-DECREASING MECHANISM FOR SCHIZOPHRENIA?

**DOI:** 10.1093/schbul/sby014.149

**Published:** 2018-04-01

**Authors:** Kristine Engemann, Carsten Bøcker Pedersen, Constantinos Tsirogiannis, Preben Bo Mortensen, Jens-Christian Svenning

**Affiliations:** Aarhus University

## Abstract

**Background:**

Schizophrenia risk has been linked to urbanization but the underlying mechanistic link remains unknown. Less green space in urbanized areas, where schizophrenia risk is high, could point to green space as an important factor. Green space is hypothesized to positively influence mental health and could mediate schizophrenia risk through noise and particle pollution removal, stress relief or other unknown mechanisms. However, the effect of green space on schizophrenia risk has not been disentangled from that of urbanization and it is unclear if different measures of green space associate differently with risk.

**Methods:**

We used satellite data from the Landsat program to quantify green space for Denmark in 30 × 30m resolution for the years 1985–2013. The effect of quantity and heterogeneity of green space and urbanization at place of residence on schizophrenia risk was estimated using cox regression from a longitudinal population-based sample of the Danish population (943 027 persons). Schizophrenia risk was controlled for a range of individual and socioeconomic characteristics that may confound the effect of green space including age, sex and parental education, salary, and employment status.

**Results:**

Living at the lowest amount of green space was associated with a 1.52-fold increased risk of developing schizophrenia compared to persons living at the highest level of green space. This association remained after adjusting for known risk factors for schizophrenia: urbanization, age, sex, and socioeconomic status. The strongest protective association was observed during the earliest childhood years and closest to place of residence.

**Discussion:**

We found green space to decrease schizophrenia risk independent of urbanization - consequently pointing to green space as a new environmental risk factor for schizophrenia development. This study supports findings from other studies highlighting the natural environment as an important factor for human health, and points to a new methodological framework that combines epidemiological studies with big data approaches.

